# Differential effects of Vav‐promoter‐driven overexpression of BCLX and BFL1 on lymphocyte survival and B cell lymphomagenesis

**DOI:** 10.1111/febs.14426

**Published:** 2018-03-24

**Authors:** Selma Tuzlak, Manuel D. Haschka, Anna‐Maria Mokina, Thomas Rülicke, Suzanne Cory, Verena Labi, Andreas Villunger

**Affiliations:** ^1^ Division of Developmental Immunology Biocenter Medical University of Innsbruck Austria; ^2^ Institute of Laboratory Animal Science University of Veterinary Medicine Vienna Austria; ^3^ Molecular Genetics of Cancer Division The Walter and Eliza Hall Institute of Medical Research Melbourne Vic. Australia; ^4^ Department of Medical Biology The University of Melbourne Vic. Australia

**Keywords:** apoptosis, BCLX, BFL1/A1, lymphomagenesis, MYC, Vav‐promoter

## Abstract

Overexpression of BCLX and BFL1/A1 has been reported in various human malignancies and is associated with poor prognosis and drug resistance, identifying these prosurvival BCL2 family members as putative drug targets. We have generated transgenic mice that express human BFL1 or human BCLX protein throughout the haematopoietic system under the control of the *Vav* gene promoter. Haematopoiesis is normal in both the *Vav‐BFL1* and *Vav‐BCLX* transgenic (TG) mice and susceptibility to spontaneous haematopoietic malignancies is not increased. Lymphoid cells from *Vav‐BCLX* TG mice exhibit increased resistance to apoptosis *in vitro* while most blood cell types form *Vav‐BFL1* TG mice were poorly protected. Both transgenes significantly accelerated lymphomagenesis in *Eμ‐MYC* TG mice and, surprisingly, the *Vav‐BFL1* transgene was the more potent. Unexpectedly, expression of transgenic BFL1 RNA and protein is significantly elevated in B lymphoid cells of *Vav‐BFL1/Eμ‐MYC* double‐transgenic compared to *Vav‐BFL1* mice, even during the preleukaemic phase, providing a rationale for the potent synergy. In contrast, *Vav‐BCLX* expression was not notably different. These mouse models of BFL1 and BCLX overexpression in lymphomas should be useful tools for the testing the efficacy of novel human BFL1‐ and BCLX‐specific inhibitors.

**Abbreviations**
AV: Annexin V
DT: double‐transgenic
Eµ‐MYC: c‐MYC gene driven by the IgH enhancer
Glc: glucocorticoids
STS: staurosporine
TG: transgene/transgenic

## Introduction

The physiological roles of BFL1/A1, an antiapoptotic member of the BCL2 family, are still poorly understood. A1 was discovered in 1993 as an early response gene in GM‐CSF‐treated bone marrow‐derived macrophages 1, 2 and later shown to be induced by antigen‐mediated activation in T and B cells 3, 4. In mice, A1 is produced by three independent genes (*Bcl2a1‐a*,* Bcl2a1‐b* and *Bcl2a1‐d*) 5 and is mainly expressed in the haematopoietic system where it is dynamically regulated in response to antigens or inflammatory cues engaging NF‐kB 6, NF‐AT 7, and PU.1 8 transcription factors. Mice that lack all functional *Bcl2a1* genes do not exhibit major impairments in the development and composition of their immune system 9 or T cell‐mediated immune responses 10. The human homologue*, BFL1*, which is highly homologous to A1 (72% amino acid identity) is encoded by a single gene 11. Elevated *BFL1* expression has been associated with many malignancies, including acute lymphoblastic leukaemia, chronic lymphocytic leukaemia and melanoma skin cancer 12, 13. In mouse models, lentiviral transduction of bone marrow cells with *Bcl2a1‐a* led to the development of B cell lymphomas in recipient mice 14 and cotransduction with human *BFL1* and *c‐MYC* caused acute myelogenous leukaemia 15. Importantly, BFL1 mutants that escape ubiquitin‐mediated proteasomal degradation are more stable and accelerate tumour formation in the presence of a dominant negative, truncated version of *p53* ^*DD*^ 16, indicating the importance of BFL1 expression levels for facilitating tumour formation.

BCLX is essential during early development as *Bclx*‐deficient mice die at embryonic day 13 due to defective erythropoiesis and massive cell death in the central nervous system 17, 18. In the haematopoietic system, BCLX deficiency leads to a loss of pre‐B cells 19, impaired erythropoiesis 20 and decreased platelet life span 21. Interestingly, although highly expressed in CD4^+^CD8^+^ double‐positive (DP) thymocytes, *Bclx* deletion does not substantially influence T cell development but only reduces the life span of DP thymocytes *ex vivo* 22, 23.

The transcription factor c‐MYC is a key transcriptional regulator involved in many cellular processes including metabolism, cell cycle and apoptosis 24. Aberrant expression of MYC is associated with a significant number of human malignancies 25, including human Burkitt's lymphomas, which harbour chromosome translocations linking the *MYC* gene with Ig heavy (*IGH*) or Ig light chain (*IGL*) loci 26. *Eμ‐MYC* transgenic mice, which model Burkitt's lymphoma to a certain degree, develop monoclonal pro‐/pre‐B and immature B cell lymphomas between 4 and 7 months of age 27, 28. Of note, *BCL2* 29, 30, *MCL1* 31 or *BCLX* 32 transgenes significantly accelerate lymphomagenesis in *Eμ‐MYC* mice, indicating the importance of overcoming apoptosis for MYC‐driven lymphomagenesis.

Little is known about the lymphomagenic potential of BFL1/A1. Using an shRNA‐based model to knock down A1 protein expression in mice, we recently observed that MYC‐induced lymphomas select against low A1 levels and that diminished A1 renders premalignant cells more susceptible to apoptosis *ex vivo* 33. Studies using mice totally lacking A1 also suggest that A1 contributes to tumour cell survival in the context of MYC overexpression 34. Moreover, the recent report of a patient with DLBCL 35 having a *BFL1/IgH* translocation as well as a MYC/*IgL* translocation suggests that BFL1 overexpression can act as a second hit in MYC‐driven B cell lymphomagenesis.

To investigate the impact of pan‐haematopoietic overexpression of BFL1 and BCLX, we have generated *Vav‐BFL1* TG and *Vav‐BCLX* TG mice. We found that both the *Vav‐BFL1* and the *Vav‐BCLX* transgenes can accelerate *Eμ‐MYC*‐driven lymphomagenesis and observed an unexpected interrelationship between MYC and BFL1 TG expression levels.

## Results

### Enforced expression of BFL1 or BCLX does not perturb haematopoiesis in mice

**Figure 1:**
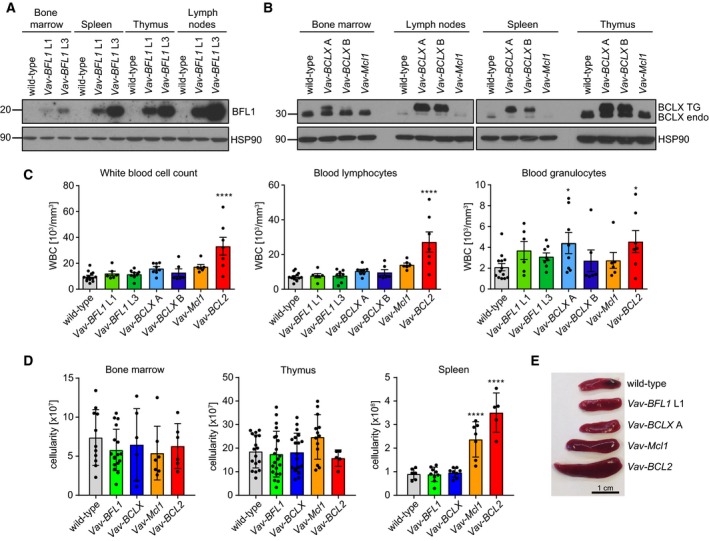
Characterization of transgene expression and composition of haematopoietic organs in *Vav‐BFL1* and *Vav‐BCLX* TG mice. (A) Bone marrow, spleen, thymus and lymph nodes were isolated from 8–12‐week‐old wild‐type, *Vav‐BFL1* (L1) and *Vav‐BFL1* (L3) mice, respectively, and processed for western blotting using anti‐BFL1‐ and anti‐HSP90‐specific antibodies. (B) Bone marrow, lymph nodes, spleen and thymus were isolated from wild‐type, *Vav‐BCLX* (A), *Vav‐BCLX* (B) or *Vav‐Mcl1* mice and processed for western analysis using anti‐BCLX‐ and anti‐HSP90‐specific antibodies. (C) Peripheral blood was sampled from mice of the indicated genotypes and white blood cell counts were determined by using a ScilVet abc blood counter (left bar graph). WBCs were further characterized as either lymphocytes (middle bar graph) or granulocytes (right bar graph). (D) Cell counts were determined from bone marrow (both femurs, left bar graph), thymus (middle bar graph) and spleen‐derived single‐cell suspensions (right bar graph). Data from *Vav‐BFL1* TG line L1 and L3 and from *Vav‐BCLX* TG line A and line B were comparable and pooled for easier representation. (E) Representative spleen specimens from wild‐type, *Vav‐BFL1* line L1*, Vav‐BCLX* line A, *Vav‐Mcl1* and *Vav‐BCL2* mice. Statistical analysis was performed using one‐way ANOVA with Dunnett's multiple comparison. *P < 0.05; ****P < 0.0001; *n* ≥ 4 ± SD.

The *BFL1* TG and *BCLX* TG mice were generated by pronuclear injection of oocytes using a haematopoietic‐specific transgenic vector driven by the *Vav* gene promoter 36. For each transgene, independent colonies were established from three PCR‐positive founders and the two lines showing detectable exogenous protein expression were chosen for further characterization (Fig. 1A,B), alongside previously derived *Vav‐Mcl1* TG 31 and *Vav‐BCL2* TG mice 37. The *Vav‐BFL1* TG and *Vav‐BCLX* TG mice were healthy, showed normal fertility and did not exhibit any premature deaths within the first year of age, unlike *Vav‐Mcl1* or *Vav‐BCL2* transgenic mice, which develop auto‐immune and/or malignant disease 31, 37, 38.

To assess the impact of transgene expression on the haematopoietic compartment, adult mice were analysed between 8 and 12 weeks of age. First, we monitored the white blood cell (WBC) counts in the peripheral blood (PB). Different to *Vav‐BCL2* TG mice neither *Vav‐BCLX* TG nor *Vav‐BFL1* TG mice had significantly increased WBC numbers in the PB (Fig. 1C). Furthermore, neither *Vav‐BFL1* nor *Vav‐BCLX* TG strains showed aberrant cellularity in bone marrow, thymus or spleen (Fig. 1D, TG lines were pooled to simplify data presentation), while *Vav‐Mcl1* and *Vav‐BCL2* TG mice showed splenomegaly (Fig. 1E), as reported before 31, 37.

**Figure 2:**
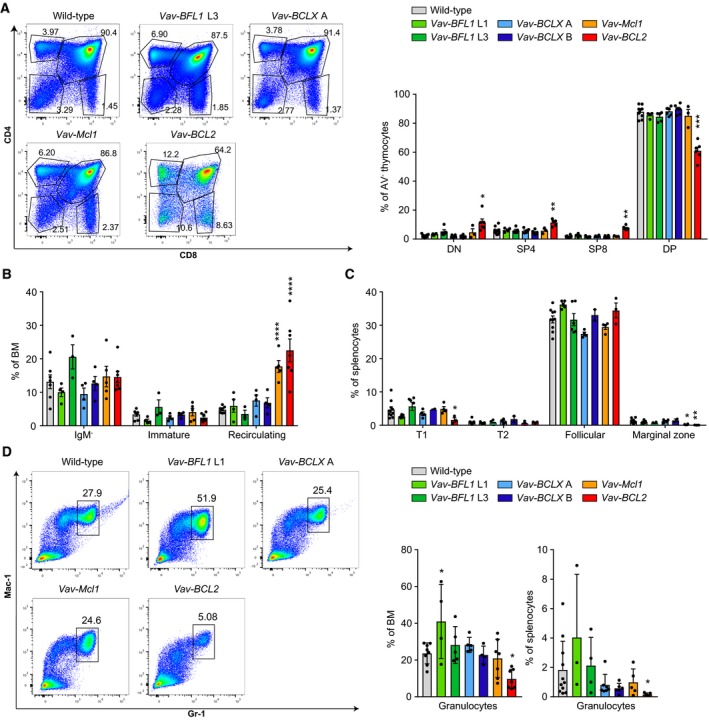
Leukocyte subset composition in *Vav‐BFL1* and *Vav‐BCLX* TG mice. (A) Representative dot‐plots of thymocytes from wild‐type, *Vav‐BFL1* TG line L3, *Vav‐BCLX* TG line A, *Vav‐Mcl1* TG and *Vav‐BCL2* TG mice stained with antibodies specific for CD8 or CD4; bar graph summarizing the results (*n* = 3–4/genotype). (B) Flow cytometry was used to assess the distribution of B220^lo^IgM^−^ pro‐/pre‐B cells, B220^lo^IgM^+^IgD^−^ immature B cells, and B220^hi^IgM^+^IgD^+^ recirculating B cells in the bone marrow (BM). (C) Splenocytes were analysed for the presence of B220^+^CD23^−^CD21^lo^IgM^+^ transitional 1 (T1) B cells, B220^+^CD23^+^CD21^hi^IgM^hi^ transitional 2 (T2) B cells, B220^+^CD23^+^CD21^+^IgM^+^ follicular B cells, and B220^+^CD23^−^CD21^hi^IgM^hi^ marginal zone B cells. (D) Representative dot‐plots of bone marrow from wild‐type, *Vav‐BFL1*,* Vav‐BCLX*,* Vav‐Mcl1* and *Vav‐BCL2* TG mice showing CD11b/Mac‐1^+^Gr‐1^hi^ granulocytes. Bar graphs: quantification of the granulocytes in the bone marrow (left graph) and spleen (right graph). Statistical analysis was performed by using one‐way ANOVA with Dunnett's multiple comparison. *P < 0.05; **P < 0.01; ***P < 0.001; ****P < 0.0001; *n* ≥ 3 ± SD.

Next, we examined the abundance of different lymphocyte subsets in primary and secondary lymphoid organs. Thymocyte development was normal in *Vav‐BFL1* and *Vav‐BCLX* TG mice throughout all developmental stages (Fig. 2A), in contrast to *Vav‐BCL2* TG mice which had decreased CD4^+^CD8^+^ DP thymocytes and increased CD4^−^CD8^−^ double negative (DN) and CD4^+^ and CD8^+^ single‐positive (SP) cells, as reported previously 37. Furthermore, the composition of mature CD4^+^ and CD8^+^ T cells in the periphery was similar between all genotypes analysed (data not shown).

Regarding B cell development, *Vav‐BFL1* and *Vav‐BCLX* TG mice did not show any major abnormalities in the bone marrow and spleen (Fig. 2B,C), unlike *Vav‐BCL2* TG and *Vav‐Mcl1* TG mice, which showed changes expected from previous studies 31, 37. One *Vav‐BFL1* TG line (L3) displayed a trend towards an increase in immature B cells and a reduction in recirculating B cells (Fig. 2B), but this did not reach statistical significance nor was it seen in the second transgenic line. In the spleen, *Vav‐BFL1* TG mice (L1) showed a tendency towards a loss of Transitional (T) 1 B cells, similar to what could be seen for *Vav‐BCL2* mice (Fig. 2C), and a mild increase in follicular B cells (Fig. 2C).

We also examined the abundance of Mac‐1^+^Gr‐1^hi^ granulocytes in the bone marrow and spleen. While the L1 *Vav‐BFL1* TG line tended to have an increased percentage of granulocytes in the bone marrow, this was not observed in the L3 line. Granulocyte numbers were also normal in *Vav‐BCLX* and *Vav‐Mcl1* TG mice (Fig. 2D), whereas *Vav‐BCL2* TG mice had a significantly lower percentage of granulocytes in the bone marrow and spleen, as noted before 37.

### Exogenous BFL1 is a weak antagonist of apoptosis

**Figure 3:**
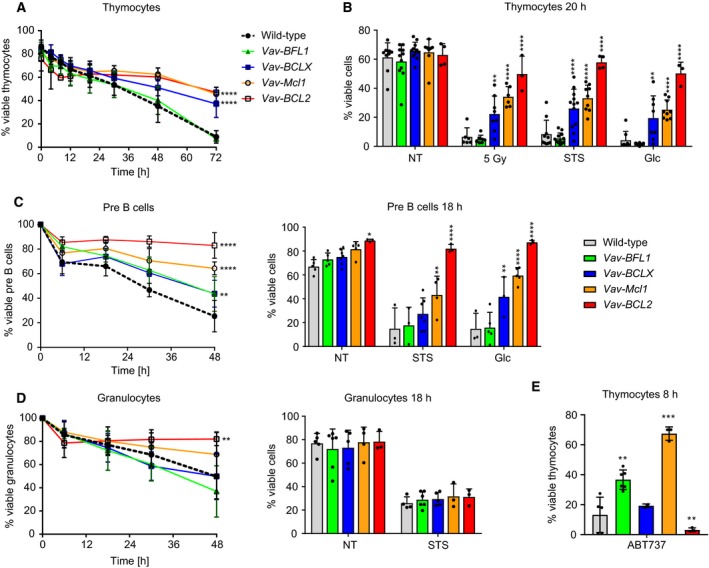
Transgenic BCLX protects lymphocytes more potently from apoptosis than BFL1. (A) Thymocytes from wild‐type, *Vav‐BFL1* (both TG lines pooled), *Vav‐BCLX* (both TG lines pooled), *Vav‐Mcl1* and *Vav‐BCL2* TG mice were cultured *in vitro* for 72 h and apoptosis was assessed over time by flow cytometry. Cells negative for Annexin V and 7AAD were considered viable. (B) Thymocytes from wild‐type, *Vav‐BFL1* (both lines pooled), *Vav‐BCLX* (both lines pooled), *Vav‐Mcl1* and *Vav‐BCL2* TG mice were either left untreated or exposed to 5 Gy γ‐irradiation, 100 nm staurosporine (STS) or 625 nm of the glucocorticoid (Glc), corticosterone respectively. Apoptosis was assessed after 20 h by flow cytometric analysis. (C) CD19^+^B220^lo^IgM^−^CD25^+^ pre‐B cells from the same mice were sorted by FACS from bone marrow and cultured for 48 h. Spontaneous apoptosis was assessed over time (left graph). In addition, sorted pre‐B cells were either left untreated or treated with 100 nm staurosporine (STS) or 625 nm corticosterone (Glc), respectively and apoptosis was assessed after 18 h by flow cytometry (right graph). (D) Gr‐1^+^ Granulocytes from mice of the indicated genotypes were sorted from the bone marrow and cultured for 48 h. Spontaneous apoptosis was assessed over time (left graph). Additionally, sorted granulocytes were cultured for 18 h either in the absence or presence of STS and apoptosis was assessed by flow cytometry (right graph). (E) Thymocytes were cultured *in vitro* for 8 h in the presence of 5 μm ABT‐737 and thereafter analysed for Annexin V and 7AAD positivity by flow cytometry. Statistical analysis was performed by using a two‐way ANOVA with Dunnett's multiple comparison. *P < 0.05; **P < 0.01; ***P < 0.001; ****P < 0.0001; *n* ≥ 3 ± SD.

Next, we analysed the ability of the overexpressed proteins to protect cells from spontaneous and drug‐induced apoptosis. Equal numbers of mice from both lines of *Vav‐BFL1* TG and *Vav‐BCLX* TG strains were analysed. No significant differences were detected and the data have therefore been pooled in Fig. 3. First, thymocytes were cultured for 4, 8, 12, 20, 30, 48 and 72 h, respectively, and analysed for Annexin V and 7AAD staining by flow cytometry. As shown previously 31, 37, *Vav‐BCL2* and *Vav‐Mcl1* TG thymocytes were strongly protected from spontaneous apoptosis (Fig. 3A). Thymocytes from both *Vav‐BCLX* TG lines also showed significantly delayed cell death, although not to the same extent, but cells from *Vav‐BFL1* TG mice remained as sensitive as wild‐type controls (Fig. 3A). A similar picture was observed upon treatment with various apoptosis‐inducing stimuli, including irradiation, staurosporine (STS) or the glucocorticoid (Glc) corticosterone.

Next we analysed pre‐B cells isolated by flow cytometry from bone marrow. Interestingly, *Vav‐BFL1* and *Vav‐BCLX* TG pre‐B cells both showed significantly delayed spontaneous apoptosis when cultured *in vitro* (Fig. 3C), albeit not to the same degree as either *Vav‐Mcl1* or *Vav‐BCL2* TG pre‐B cells (Fig. 3C). However, although the *Vav‐BCLX TG* also protected pre‐B cells from STS and Glc‐induced apoptosis*, Vav‐BFL1* expression did not (Fig. 3C, bar graph).

Neither the *Vav‐BFL1* nor the *Vav‐BCLX* TG significantly enhanced the overall viability of bone marrow granulocytes *in vitro*, while *Vav‐Mcl1* and especially *Vav‐BCL2* TG expression did so effectively (Fig. 3D). Strikingly, none of the prosurvival proteins was able to protect granulocytes from STS‐induced cell death at the time‐point chosen for analysis (Fig. 3D bar graph).

Lastly, we treated thymocytes with ABT‐737, a BH3 mimetic that induces apoptosis by inhibiting BCL2, BCLX and BCLW. Confirming the functionality of the transgene, *Vav‐BFL1* TG thymocytes were significantly protected from ABT‐737‐induced apoptosis, although the protection was not as pronounced as that seen in *Vav‐Mcl1* TG thymocytes (Fig. 3E). As expected, thymocytes from *Vav‐BCLX* and *Vav‐BCL2* mice were highly sensitive to ABT‐737.

### Quantitative rather than qualitative differences in between different BCL2‐prosurvival proteins define the degree of protection from thymocyte apoptosis

**Figure 4:**
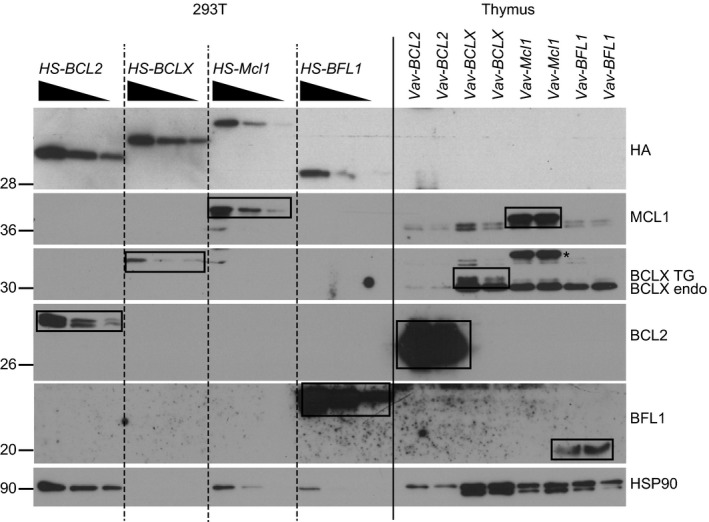
Comparative analysis reveals substantial differences in transgene expression levels across different *Vav*‐TG mice. 293T cells were transiently transfected with plasmids encoding HA/Streptavidin‐tagged BCL2, BCLX, MCL1 and BFL1 respectively. Comparable amounts of HA‐tagged proteins were loaded in different dilution next to 20 μg total thymocyte extract from *Vav‐BCL2, Vav‐BCLX, Vav‐Mcl1* and *Vav‐BFL1* TG mice respectively. Protein levels of HA‐tagged and transgenic BFL1, MCL1, BCLX, BCL2 and, for reference, HSP90 were assessed by western blotting.

We wished to clarify whether the degree of protection correlated with the level of transgene expression rather than with qualitative functional differences between the prosurvival proteins. To assist in the quantitation, we prepared transiently transfected 293T cells expressing *cDNAs* encoding for an HA and streptavidin (HS)‐tagged version of *BCL2, BCLX, Mcl1* and *BFL1* and loaded different amounts of these proteins next to 20 μg total protein lysates from the TG thymocytes (Fig. 4). Since the quantity of the HS‐tagged proteins were within a comparable range within the lysates from transfected 293T cells, as shown by the HA western blot, we were able to better judge the relative TG expression found in thymocytes using target‐specific antibodies. This comparison made it evident that the *Vav‐BCL2* TG was expressed at much higher levels in the thymus when compared to the highest concentration of 293T lysate loaded. In contrast, the *Vav‐BCLX* and *Vav‐Mcl1* TG thymocyte extracts showed comparable signals to those seen in the highest concentration of 293T cell lysate loaded. This suggests that the relative expression levels achieved in *Vav‐BCLX* and *Vav‐Mcl1* TG thymocytes were comparable to each other but much lower than those of BCL2 in *Vav‐BCL2* TG thymocytes. Strikingly, the BFL1 signal from the thymocyte lysates of *Vav‐BFL1* TG mice was lower than the lowest signal generated from the *HS‐BFL1* dilution series in 293T cell extracts. We conclude that thymocytes from *Vav‐BCL2* TG mice express the highest amounts of transgenic protein, those from *Vav‐BCLX* and *Vav‐Mcl1* TG express lower levels, albeit comparable to each other, and those from *Vav‐BFL1* TG mice have the lowest expression. These results partly explain the differences observed in the relative resistance conveyed by the different transgenes to apoptosis‐inducing stimuli in culture.

### *BFL1*‐*MYC* crosstalk accelerates B‐cell lymphomagenesis in mice

**Figure 5:**
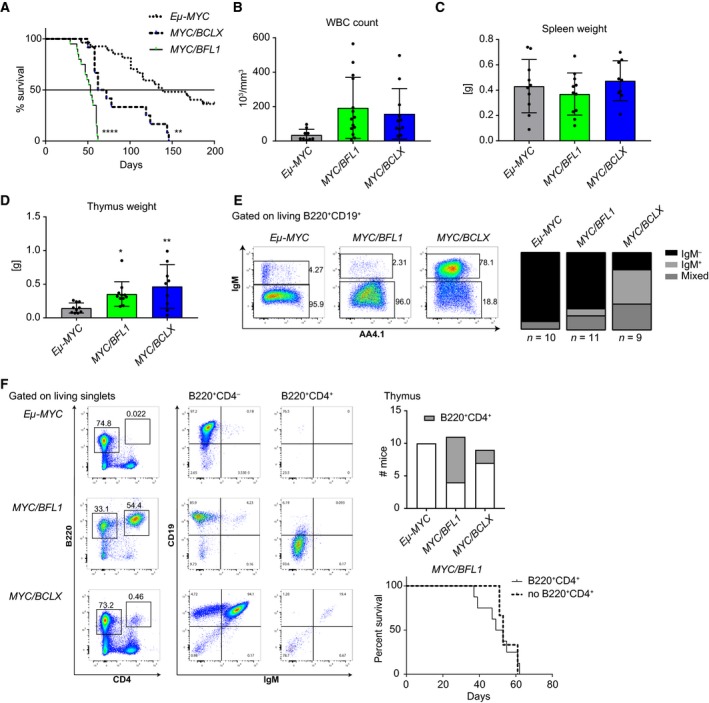
*Vav‐BFL1* and *Vav‐BCLX* transgenes accelerate lymphomagenesis in *Eμ‐Myc* mice. (A) Kaplan–Meier plot of MYC‐induced lymphomagenesis in *Eμ‐MYC* TG (*n* = 27), *Eμ‐MYC/Vav‐BFL1* (L3 and L1 pooled, *n* = 17) and *Eμ‐MYC/Vav‐BCLX* (A, *n* = 11) DT mice. (B) White blood cell counts (WBC) were analysed in tumour‐bearing mice. Thereafter, mice were sacrificed and (C) spleen and (D) thymus weights were assessed. (E) Representative dot‐plots of the tumour phenotype found in the spleen. Overall tumour phenotype quantification (see Tables S1–S3) is shown on the right. IgM^−^ refers to CD19^+^B220^+^IgM^−^ lymphoma cells, IgM^+^ refers to CD19^+^B220^+^IgM^+^ lymphoma cells, mixed refers to lymphomas containing both, CD19^+^B220^+^IgM^−^ and CD19^+^B220^+^IgM^+^ tumour cell types (F) Representative dot‐plots of stem/progenitor cell lymphomas in the thymus. Other than B220^+^CD4^−^ lymphoma cells B220^+^CD4^+^ lymphoma cells do not express CD19. Bar graph: Quantification of CD19^−^B220^+^CD4^+^ stem/progenitor cell lymphomas in the thymus. Kaplan–Meier plot: Survival curve of *Eμ‐MYC/Vav‐BFL1* DT mice with or without B220^+^CD4^+^ stem/progenitor cell lymphomas in their thymus. Statistical analysis for tumour‐free survival was performed by using a log‐rank (Mantel–Cox) test. All other statistical analyses were performed by using a one‐way ANOVA with Holm‐Sidak's multiple comparison test compared to *Eμ‐MYC* TG. *P < 0.05; **P < 0.01; ****P < 0.0001; *n* ≥ 9 ± SD.

Since the *Vav‐BCL2* and *Vav‐Mcl1* transgenes accelerated *Eμ‐MYC*‐driven lymphomagenesis 30, 31, we aimed to test if this was also the case for *Vav‐BCLX* TG and *Vav‐BFL1* TG mice. Therefore, *Vav‐BCLX* TG mice (line A) and *Vav‐BFL1* TG lines 1 and 3 mice (data pooled) were intercrossed with *Eμ‐MYC* TG mice and monitored for acute signs of lymphomagenesis such as enlarged lymph nodes and shortness of breath. Of note, the *Eμ‐MYC/Vav‐BFL1* double‐transgenic (DT) mice succumbed significantly faster to malignancy (median survival 53 days) than *Eμ‐MYC/Vav‐BCLX* DT mice (median survival 67 days) and *Eμ‐MYC* TG mice (median survival 139 days) (Fig. 5A). This result was unexpected since we had observed comparable protective capacity of the *Vav‐BFL1* and *Vav‐BCLX* transgenes in cultured pre‐B cells (Fig. 3C) and significantly better protection by the *Vav‐BCLX* transgene against glucocorticoid treatment. Sick *Eμ‐MYC/Vav‐BFL1* and *Eμ‐MYC/Vav‐BCLX* DT mice had similar WBC counts and these were in both cases significantly higher than that for sick *Eμ‐MYC* TG mice (Fig. 5B). Spleen weights were comparable between all three genotypes (Fig. 5C) while thymus weights were significantly higher in both *Eμ‐MYC/Vav‐BFL1* and *Eμ‐MYC/Vav‐BCLX* DT mice than in *Eμ‐MYC* TG mice (Fig. 5D). Next, we analysed the phenotype of the tumours by flow cytometric analysis (Fig 5E, Tables S1–S3). The *Eμ‐MYC* mice developed mainly immature B220^+^CD19^+^IgM^−^ tumours (Table S1). Most *Eμ‐MYC/Vav‐BFL1* mice also developed pre‐B lymphomas (Fig. 5E, Table S2) but a significant number (7 of 11 mice analysed) additionally developed CD19^−^B220^+^CD4^+^ progenitor cell lymphomas that have been described before for *Eμ‐MYC/Eμ‐BCL2* mice 29. In the *Eμ‐MYC/Vav‐BFL1* mice, these progenitor tumours were largely restricted to the thymus (Fig. 5F, Table S2). However, the onset of disease was not significantly different between *Eμ‐MYC/Vav‐BFL1* mice that harboured CD19^−^B220^+^CD4^+^ progenitor cell lymphomas in their thymus and those that did not (Fig. 5F, Kaplan–Meier plot). Interestingly, *Eμ‐MYC/Vav‐BCLX* DT tumours appeared more variable by showing IgM^−^, IgM^+^ and mixed phenotypes consisting of IgM^+^ and IgM^−^ lymphoma cells respectively (Fig. 5E and Table S3). However, the different tumour types did not differ in the onset of disease (not shown).

**Figure 6:**
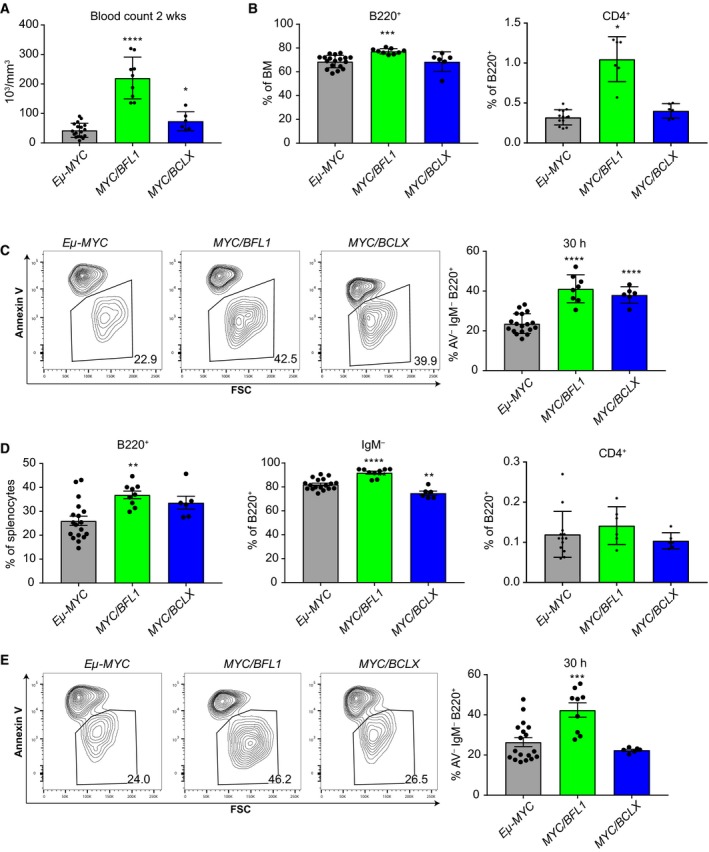
BFL1 overexpression protects premalignant immature B cells from MYC‐induced apoptosis. (A) White blood cell counts from 2–week‐old premalignant *Eμ‐MYC, Eμ‐MYC/Vav‐BFL1* (L1 and L3 pooled), and *Eμ‐MYC/Vav‐BCLX* (line A) mice were assessed. (B) Percentage of B220^+^ B lymphoid cells in the bone marrow was analysed by flow cytometry (left bar). B220^+^ cells were further discriminated into CD19^−^CD4^+^ cells (right bar). (C) Total bone marrow was cultured for 30 h and the abundance of living (Annexin V^−^) B220^+^IgM^−^ immature B lymphoid cells was assessed by flow cytometry. (D) Abundance of total B220^+^ B lymphoid cells in the spleen was analysed by flow cytometry (left graph). B220^+^ cells were further discriminated into CD19^+^IgM^−^ (middle graph) and CD19^−^CD4^+^ cells (right graph). (E) Total splenocytes were cultured for 30 h and abundance of living (Annexin V^−^) B220^+^IgM^−^ immature B lymphoid cells was assessed by flow cytometry. Statistical analysis was performed by using a one‐way ANOVA with Dunnett's multiple comparison test compared to *Eμ‐MYC* control. *P < 0.05; **P < 0.01; ***P < 0.001; ****P < 0.0001; *n* ≥ 3 ± SD.

Next, we compared the premalignant phenotypes of *Eμ‐MYC/Vav‐BFL1* and *Eμ‐MYC/Vav‐BCLX* DT mice. As *Eμ‐MYC/Vav‐BFL1* DT mice can succumb to tumours as early as 29 days, we analysed the mice at 2 weeks of age in order to avoid transformed cells. Importantly, we analysed both *Eμ‐MYC/Vav‐BFL1* lines to exclude potential side‐effects caused by random transgene insertion. First, we monitored white blood cell counts. Pups from both *Eμ‐MYC/Vav‐BFL1* DT lines had five times higher WBC counts than *Eμ‐MYC* TG pups at that age (Fig. 6A). Interestingly, WBC counts from *Eμ‐MYC/Vav‐BCLX* DT pups although significantly higher than those of *Eμ‐MYC* TG mice were three times lower than in *Eμ‐MYC/Vav‐BFL1* DT pups (Fig. 6A). Of note, the elevated WBC counts represent an early burst of pre‐B cells, caused by MYC overexpression 39, and the WBC counts subsided by 4 weeks of age (data not shown). Cell counts from the bone marrow and spleen of 2‐week‐old mice were comparable between all the genotypes (data not shown). However, the percentage of B220^+^ B lymphoid cells in the bone marrow was significantly higher in *Eμ‐MYC/Vav‐BFL1* DT mice than in *Eμ‐MYC* TG and *Eμ‐MYC/Vav‐BCLX* DT mice (Fig. 6B left graph). Most B220^+^ cells were immature CD19^+^IgM^−^ cells (data not shown), but a small proportion also represented CD19^−^CD4^+^ cells and was significantly higher in *Eμ‐MYC/Vav‐BFL1* DT mice compared to the other genotypes (Fig. 6B right graph). However, this population was present in comparably low numbers (0.1% of all B220^+^ cells) in the blood (data not shown) and the spleen (Fig. 6D right graph) of all tested genotypes. Importantly, B220^+^ B lymphoid cells from *Eμ‐MYC/Vav‐BFL1* and *Eμ‐MYC/Vav‐BCLX* DT bone marrows survived better in culture than those from *Eμ‐MYC* mice (Fig. 6C). B220^+^ B lymphoid cells were also increased proportionally in the spleen of both DT genotypes compared to *Eμ‐MYC* TG mice, although this reached significance only for *Eμ‐MYC/Vav‐BFL1* DT mice (Fig. 6D left graph). Interestingly, *Eμ‐MYC/Vav‐BFL1* DT mice had significantly more B220^+^CD19^+^IgM^−^ B lymphoid cells in the spleen than *Eμ‐MYC* TG mice, and *Eμ‐MYC/Vav‐BCLX* DT mice showed significantly less (Fig. 6D middle graph) but no increase in B220^+^CD19^−^CD4^+^ cells (Fig. 6D right graph). Furthermore, only the *Eμ‐MYC/Vav‐BFL1* splenic B lymphoid cells showed increased survival in culture (Fig. 6E).

### MYC overexpression increases transgenic BFL1 RNA and protein levels

**Figure 7:**
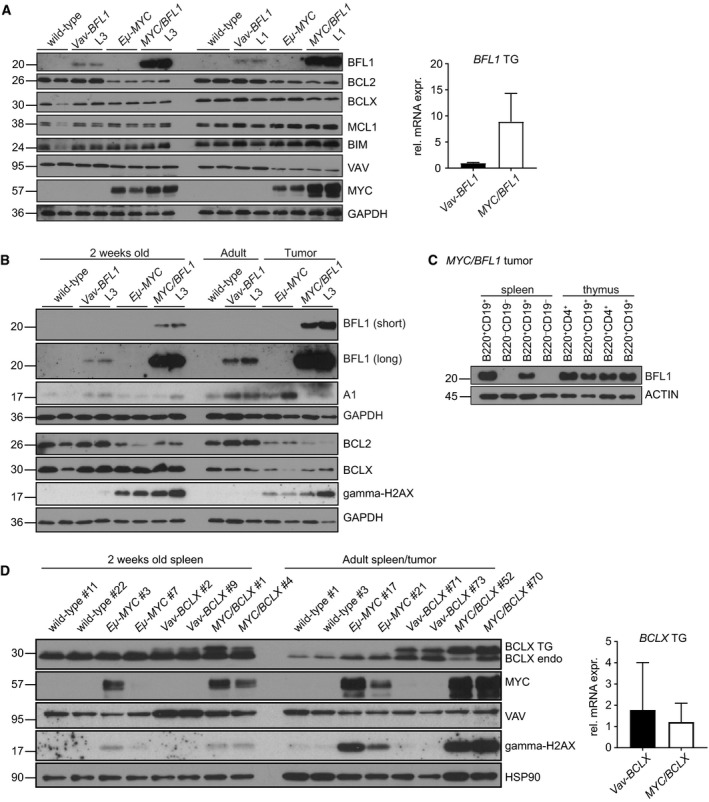
MYC elevates BFL1 mRNA and protein levels (A) Spleens were dissected from 2‐week‐old wild‐type, *Vav‐BFL1* (L3) TG, *Eμ‐MYC* TG, *Eμ‐MYC/Vav‐BFL1* (L3) DT, *Vav‐BFL1* (L1) TG and *Eμ‐MYC/Vav‐BFL1* (L1) DT mice and, following erythrocyte depletion, cells were lysed in NP‐40 containing buffer for western analysis using antibodies specific for the indicated proteins. mRNA was isolated from spleens from 2‐week‐old *Vav‐BFL1* (L3) TG and *Eμ‐MYC/Vav‐BFL1* (L3) DT mice, transcribed into cDNA and assessed for *BFL1* expression levels. (B) Spleens from 2‐week‐old wild‐type, *Vav‐BFL1* (L3) TG, *Eμ‐MYC* TG and *Eμ‐MYC/Vav‐BFL1* (L3) DT mice as well as from adult wild‐type and *Vav‐BFL1* (L3) TG and tumour‐bearing *Eμ‐MYC* TG and *Eμ‐MYC/Vav‐BFL1* (L3) DT mice were isolated and erythrocyte‐depleted cell preparations were lysed in NP‐40 containing lysis buffer. Western blots were probed for indicated proteins. (C) B220^+^CD19^+^ B lymphoid and B220^−^CD19^−^ non‐B lymphoid cells were isolated by FACS from spleens of tumour‐bearing *Eμ‐MYC/Vav‐BFL1* DT mice. Furthermore, B220^+^CD19^+^ and B220^+^CD19^−^CD4^+^ cells, respectively, were isolated from thymocytes of tumour‐bearing *Eμ‐MYC/Vav‐BFL1* DT mice. Western blot analysis was performed as described in (A). (D) Spleens from 2‐week‐old wild‐type, *Vav‐BCLX* (line A) TG, *Eμ‐MYC* TG and *Eμ‐MYC/Vav‐BCLX* (A) DT mice as well as from adult wild‐type and *Vav‐BCLX* (A) TG and tumour‐bearing *Eμ‐MYC* TG and *Eμ‐MYC/Vav‐BCLX* (line A) DT mice were isolated and erythrocyte‐depleted cells were lysed in NP‐40 containing lysis buffer. Western blots were probed for the indicated proteins using specific antibodies. mRNA was isolated from spleens from 2‐week‐old *Vav‐BCLX* (line A) TG, and *Eμ‐MYC/Vav‐BCLX* (line A) DT mice, transcribed into cDNA and assessed for *FLAG‐BCLX* expression levels.

We were surprised by the strong acceleration of disease in *Eμ‐MYC/Vav‐BFL1* DT mice and the enhanced survival capacity of premalignant *Eμ‐MYC/Vav‐BFL1* immature B cells as we had not observed major impact from expression of *Vav‐BFL1* alone. We, therefore, wondered whether *Vav‐BFL1* transgene expression had changed in the *Eμ‐MYC* TG background. Indeed, western blots revealed a striking difference in BFL1 levels in total splenocytes isolated from 2‐week‐old *Eμ‐MYC/Vav‐BFL1* DT mice compared to those from age‐matched *Vav‐BFL1* TG mice (Fig. 7A). Importantly, both transgenic lines showed highly enhanced BFL1 expression on the *Eμ‐MYC* TG background. This phenomenon did not appear to be a global event since other BCL2 family members were not affected. Furthermore, we showed that the enhanced transgene expression was not *Vav* promoter dependent since the endogenous VAV protein was not increased by MYC overexpression, but rather mildly reduced. Importantly, the increase in BFL1 expression by MYC overexpression happened to the same extent in both independently generated *Vav‐BFL1* TG lines, minimizing potential transgene insertion effects. Interestingly, *BFL1* mRNA was found increased by approximately ninefold in *Vav‐BFL1/Eμ‐MYC* DT splenocytes when compared to mRNA levels found in *Vav‐BFL1* TG mice (Fig. 7A, bar graph). Endogenous A1 expression levels were not found substantially upregulated in total splenocytes by MYC overexpression, neither on mRNA nor on protein levels (Fig. 7B and data not shown). Together these findings argue for enhanced *BFL1* mRNA stability or reduced protein turnover. MYC protein expression was also slightly higher in *Eμ‐MYC/Vav‐BFL1* DT splenocytes than in *Eμ‐MYC* TG samples (Fig. 7A) although *MYC* mRNA levels were not significantly elevated (not shown), indicative of a feed‐forward loop where increased cell death resistance allows cells to tolerate increased MYC protein levels. In tumour samples, we found that BFL1 expression was further elevated when compared to splenocytes from 2‐week‐old premalignant *Eμ‐MYC/Vav‐BFL1* DT mice (Fig. 7B). In order to determine BFL1 expression levels in the different tumour cell subsets, we FACS‐sorted B220^+^CD19^+^ tumour cells and B220^−^CD19^−^ nontumour cells from the spleen and B220^+^CD19^+^ and B220^+^CD19^−^CD4^+^ tumour cells from the thymus of diseased *Eμ‐MYC/Vav‐BFL1* DT mice. BFL1 protein levels were only detectable in B lymphoid tumour cells, while they were absent in non‐B220^+^ cells (Fig. 7C). Furthermore, we could not detect any quantitative differences in the BFL1 expression between B220^+^CD19^+^ and B220^+^CD19^−^CD4^+^ tumour populations.

Comparable increases in transgenic BCLX protein expression were not observed in splenocytes from premalignant *Eμ‐MYC/Vav‐BCLX* DT vs. premalignant *Vav‐BCLX* TG mice, although levels increased mildly in *Eμ‐MYC/Vav‐BCLX* tumours (Fig. 7D). Furthermore, *BCLX* TG mRNA was not influenced by exogenous MYC expression in 2‐week‐old spleen extracts (Fig. 7D, bar graph). We conclude that *Eμ‐MYC* overexpression positively influences expression of transgenic *Vav‐BFL1,* at both the protein and RNA level, but not the *Vav‐BCLX* transgene.

## Discussion

The physiological importance of BFL1/A1 is still poorly understood, despite increased recent efforts. In a broad variety of immune cells, including neutrophils, granulocytes, B and T cells, A1 is rapidly inducible in response to diverse stimuli, including antigen receptor stimulation, GM‐CSF, BAFF receptor or CD40‐ligation 40, 41, predictive of crucial roles in inflammation and immunity. Nevertheless, complete deficiency of A1 does not impair the normal development or function of the immune system nor does it influence the normal behaviour and life span of mice 9, 10.

Here, we describe the generation and characterization of mice that overexpress human BFL1 or human BCLX under the control of a haematopoietic‐specific vector driven by the *Vav* gene promoter 42. In contrast to *Vav‐BCL2* 37 and *Vav‐Mcl1* 31 TG mice, the overall composition and cellularity of all major lymphoid organs was essentially normal in both *Vav‐BCLX* and Vav‐*BFL1* mice (Figs 1 and 2). Interestingly, while the *Vav‐BCLX* TG partially protected both thymocytes and pre‐B cells from spontaneous and drug‐induced apoptosis, the *Vav‐BFL1* TG provided only a minor survival benefit in pre‐B cells or when thymocytes were treated with ABT‐737 (Fig. 3). In general, the graded survival benefits observed in the different mouse models correlated directly with the level of transgenic protein expressed (Figs 3 and 4). It remains unclear why both *Vav‐BFL1* TG lines express such low amounts of BFL1, especially when compared to the two *Vav‐BCLX* TG lines that were generated contemporaneously, but this likely reflects the short half‐life of BFL1 43, 44.

Surprisingly, when we crossed *Vav‐BFL1* TG with *Eμ‐MYC* TG mice, expression of the *Vav‐BFL1* TG was boosted (Fig. 7) and lymphomagenesis was dramatically accelerated (Fig. 5). Indeed, the median survival of *Eμ‐MYC/Vav‐BFL1* DT lymphomas was only 53 days, comparable to that observed for *Eμ‐MYC* mice lacking the BH3‐only protein BIM 45, 46. While the *Vav‐BCLX* transgene also accelerated lymphomagenesis in *Eμ‐MYC* mice (median survival 67 days), previously described *Eμ*‐*Bclx*/*Eμ‐MYC* DT mice died at an age of only 6 weeks (42 days) 32. The difference is likely to reflect the relative level of BCLX protein achieved.

Intriguing differences were observed in the tumour phenotypes. C57BL/6 *Eμ‐MYC* TG mice can develop either pro‐/pre‐B or B lymphomas 34, 45, with the former dominating in our colony. Intriguingly, our *Eμ‐MYC/Vav‐BCLX* DT mice developed mainly IgM^+^ B cell lymphomas, like *Eμ‐MYC* mice lacking BIM 45 or BMF 46. It has been reported that *Eμ‐MYC*‐driven IgM^−^ immature B lymphomas are more aggressive and develop faster than their IgM^+^ counterparts 30. This observation would be consistent with the prolonged tumour‐latency observed for *Eμ‐MYC/Vav‐BCLX* DT mice compared to *Eμ‐MYC/Vav‐BFL1* DT mice that only develop IgM^−^ tumours. However, within the group of *Eμ‐MYC/Vav‐BCLX* DT mice, no differences in latency were observed between the different immunophenotypes (not shown).

Interestingly, *Eμ‐MYC/Vav‐BFL1* DT mice developed mainly immature (B220^+^ IgM^−^) lymphomas, with a significant proportion (64%) that additionally developed CD19^−^B220^+^CD4^+^ stem/progenitor cell lymphomas in the thymus. Stem/progenitor cell lymphomas also dominated in *Eμ‐BCL2/Eμ‐MYC* DT, *Eμ‐Bclx/Eμ‐MYC* DT and *Vav‐Mcl1/Eμ‐MYC* DT mice 29, 31, 32, while *Vav‐BCL2/Eμ‐MYC* DT mice developed IgM^−^CD19^+^CD43^+^ pro‐B cell tumours 30. Interestingly, WBC counts were massively increased in premalignant *Eμ‐MYC/Vav‐BFL1* DT mice compared to *Eμ‐MYC* TG littermate controls or age‐matched *Eμ‐MYC/Vav‐BCLX* DT mice. Presumably the larger pool of precursor cells and their reduced susceptibility to apoptosis explains the accelerated lymphomagenesis.

Lastly, we are intrigued by the elevated expression of BFL1 protein and mRNA in premalignant *Eμ‐MYC/Vav‐BFL1* TG splenocytes compared to *Vav‐BFL1* TG samples (Fig. 7A,B). Intriguingly, BFL1 was upregulated to the same extent in both *Eμ‐MYC/Vav‐BFL1* DT lines (Fig. 7A) and was already apparent at 2 weeks of age, arguing against clonal expansion of cells with stronger *Vav‐BFL1* expression during transformation. *Eμ‐MYC/Vav‐BCLX* DT cells did not show elevated transgene expression compared to *Vav‐BCLX* TG littermates (Fig. 7D). Since MYC influences global gene expression 47, the elevation of transgenic BFL1 expression might reflect integration of the transgene within MYC accessible sites, in both independently generated *Vav‐BFL1* TG lines. It might also be possible that c‐MYC stabilizes the transgenic *BFL1* mRNA in the context of an artificial 3′ UTR as endogenous A1 was not found elevated (Fig. 7B). The molecular mechanism responsible for this intriguing phenomenon remains to be investigated further.

Together, our findings underline the major impact of elevated BFL1 on tumour development, an effect that might not be confined to MYC‐induced lymphomas. They also emphasize the potential usefulness of the development of BFL1‐specific inhibitors for cancer treatment and the mouse model described here might be perfectly suited for their preclinical testing.

## Materials and methods

### Mice

*Vav‐BCL2* 37, *Vav‐Mcl1* 31, and *Eμ‐MYC* 48 mice were described previously. *Vav‐BFL1* and *Vav‐BCLX* TG mice were generated by pronuclear injection of the *VavP* vectors 36 encoding human *FLAG‐BFL1* or human *FLAG‐BCLX cDNA* into C57BL/6N oocytes. The founder lines were designated as C57BL/6N‐Tg(*Vav‐BFL1*)676, 677, 686Biat and C57BL/6N‐Tg(*Vav‐BCLX*)670, 671, 672Biat, herein referred as *Vav‐BFL1* TG line L1, L2, L3 and *Vav‐BCLX* TG line A, B, C. To generate *Eμ‐MYC/Vav‐BFL1* or *Eμ‐MYC/Vav‐BCLX* DT offspring, *Vav‐BFL1* or *Vav‐BCLX* females were mated with *Eμ‐MYC* TG males. Tumour onset was determined by regular palpation (three times a week) and by monitoring of short breath, activity and/or scruffy fur. All mice were maintained on a C57BL/6N genetic background. All experiments were performed in accordance with Austrian legislation (BMWF‐66.011/0008‐11/3b/2014). For the production of transgenic mice an experiment license was granted under BMWF‐68.205/0258‐II/3b/2011.

### Haematopoietic cell analysis and flow cytometry

Peripheral blood was analysed with a scilVet abc blood counter (Viernheim, Germany) or by flow cytometric analysis after red blood cell lysis using 0.168 m ammonium chloride in PBS. Single‐cell suspensions were prepared from thymus, lymph nodes (axillary, brachial, inguinal and mesenteric), bone marrow (from femurs) and spleen, and viable cells were counted using a Neubauer counting chamber by trypan blue exclusion. Cell composition was determined by staining with cell surface marker‐specific antibodies and flow cytometric analysis using a LSR Fortessa (BD Biosciences, Franklin Lakes, NJ, USA). Stained single‐cell suspensions were sorted using a FACS ARIA III (BD Biosciences). The following labelled monoclonal antibodies were used: anti‐Gr‐1 (RB6‐8C5), anti‐CD11b (M1/70), anti‐B220 (RA3‐6B2), anti‐TCRβ (HS7‐597), anti‐CD4 (GK1.5), anti‐CD8 (53‐6.7), anti‐CD25 (PC61), anti‐CD44 (IM7), anti‐CD62L (MEL‐14), anti‐CD19 (6D5), anti‐CD93 (AA4.1), anti‐c‐kit (ACK2), anti‐IgM (eb121‐15F9), anti‐IgD (11‐26c.2a), anti‐CD21 (7G6), anti‐CD23 (B3B4). Cell viability was determined by using Annexin V‐FITC and 7AAD (both eBioscience, ThermoFisher Scientific, Inc., Grand Island, NY, USA). FCS files were analysed by using flowjo Version 10.4 for Windows.

### Cell culture, apoptosis assays and chemical compounds

Total thymocytes, sorted pre‐B cells or sorted granulocytes were cultured in RPMI‐1640 medium (Sigma‐Aldrich, St. Louis, MO, USA) supplemented with 10% FCS (Sigma‐Aldrich, F7524), 250 μm l‐glutamine (PAA Laboratories, Vienna, Austria, M11‐004), 100 U·mL^−1^ penicillin, 100 μg·mL^−1^ streptomycin (PAA Laboratories, P11‐010), 100 μm nonessential amino acids (Gibco, ThermoFisher Scientific, Inc., 1091607), 1 mm sodium pyruvate (Gibco, 1046485) and 50 μm β‐mercapthoethanol (AppliChem, Darmstadt, Germany) at 37 °C in a humidified atmosphere containing 5% CO_2_. Apoptosis was induced either by γ‐irradiation with 5 Gy, 100 nm staurosporine (Sigma, S6942), 625 nm corticosterone (Sigma, C‐2503) or 5 μm ABT‐737 (ApexBio, Houston, TX, USA, A8194 Batch No.1).

### Immunoblotting

For the comparative western blot analysis, 293T cells were transiently transfected with 1 μg/6‐well *pTO‐HS‐BCL2, pTO‐HS‐BCLX, pTO‐HS‐Mcl1*, and *pTO‐HS‐BFL1* constructs by using polyethylenimine 49 and harvested after 24 h. Cell pellets were lysed using NP‐40 containing lysis buffer [50 mm Tris pH 8.0, 150 mm NaCl, 0.5% NP‐40, 50 mm NaF, 1 mm Na_3_VO_4_, 1 mm PMSF, one tablet protease inhibitors (EDTA free, Roche, Basel, Switzerland) per 10 mL and 30 μg·mL^−1^ DNaseI (Sigma‐Aldrich)] and protein concentration was quantified with Bradford reagent (500‐0006, Bio‐Rad, Munich, Germany). Twenty to thirty micrograms of total protein was loaded on 12% Bis‐Tris acrylamide gels and blotted on Amersham™ Hybond™—ECL nitrocellulose membranes (GE Healthcare, Little Chalfont, UK). The following antibodies were used for protein detection: rabbit anti‐BFL1 (kindly provided by Jannie Borst 50), rat anti‐mouse A1 (WEHI, 6D6, 2 μg·mL^−1^) 51, rabbit anti‐BIM/BOD (Enzo, Farmingdale, NY, USA, polyclonal, ADI‐AAP‐330‐E, 0.2 μg·mL^−1^), rabbit anti‐MCL1 (polyclonal, Rockland, Pottstown, PA, USA, Cat# 600‐401‐394, 2.2 μg·mL^−1^), rabbit anti‐BCLX (54H6, Cell Signaling, Danvers, MA, USA, Cat# CS2764, 1 : 1000), mouse anti‐BCL2 (7/Bcl‐2, BD Biosciences, 0.5 μg·mL^−1^), mouse anti‐HA (HA.11, Covance, Princeton, NJ, USA, 1 : 1000), rabbit anti‐MYC (Y69, Abcam, Cambridge, UK, ab32072), rabbit anti‐VAV1 (Cell Signaling #2502, 1 : 1000), rabbit anti‐gamma‐H2A.X (Ser139, Cell Signaling #2577, 1 : 1000), rabbit anti‐GAPDH (14C10, Cell Signaling #2118, 1 : 5000), rabbit anti‐beta‐Actin (polyclonal, Cell Signaling #4967, 1:1000) and mouse anti‐HSP90 (F‐8, Santa Cruz, Dallas, TX, USA, Cat# sc‐13119, 0.2 μg·mL^−1^). All primary antibodies were diluted in 5% BSA in PBST and blots were incubated overnight at 4 °C.

### mRNA isolation and quantitative real‐time PCR analysis

RNA was isolated using TRIzol (ThermoFisher Scientific, Inc.) and quantified with a NanoDrop 1000 Spectrophotometer (ThermoFisher Scientific, Inc.). Two hundred nanograms of RNA was reverse transcribed into cDNA using iScript cDNA Synthesis Kit (Bio‐Rad) according to the manufacturer's instruction. Quantitative real‐time PCR was performed with a 100th part of the cDNA in a StepOnePlus System (ThermoFisher Scientific, Inc.) using Bimake SYBR Green (Bimake, Houston, TX, USA) according to the manufacturer's instructions and 100 nm of the following primers: *Flag‐BFL1* forward primer ACA AAG ACG ATG ACG ATA AAA CAG A and reverse primer AGC ACT CTG GAC GTT TTG CT; *Flag‐BCLX* forward primer: CAA AGA CGA TGA CGA TAA AGG ATC T and reverse primer TCC AGC TGT ATC CTT TCT GGG A; *Actin‐beta* forward primer: ACT GGG ACG ACA TGG AGA AG and reverse primer GGG GTG TTG AAG GTC TCA AA. PCR conditions were 95 °C for 10 min, 40 cycles of (95 °C for 15 s and 60 °C for 60 s), 95 °C for 15 s, 60 °C for 60 s followed by a melting curve with 0.3 °C increment steps up to 95 °C for 15 s. Results were normalized to *Actin‐beta* expression to be compared with the ΔΔ*C*_t_ relative quantification method.

### Statistical analysis

Statistical analysis was performed using graphpad prism Version 7.03. for Windows, GraphPad Software, La Jolla, CA, USA, http://www.graphpad.com. Used tests are indicated in the figure legends.

## Conflict of interest

The authors declare no financial conflict of interest.

## Author contributions

ST performed experiments, analysed data, wrote paper and prepared figures; MDH and AMM performed experiments and analysed data; VL provided reagents, performed experiments and discussed data; TR generated transgenic mice, SC provided transgenic mice, discussed data, revised paper; AV conceived and planned study, and wrote paper.

## Supporting information


**Table S1.** Phenotypic characterization of lymphomas arising in *Eμ‐MYC* TG mice.
**Table S2.** Phenotypic characterization of lymphomas arising in *Eμ‐MYC/Vav‐BFL1* DT mice.
**Table S3.** Phenotypic characterization of lymphomas arising in *Eμ‐MYC/Vav‐BCLX* DT mice.
