# Supplementary material for: Differential effects of Vav‐promoter‐driven overexpression of BCLX and BFL1 on lymphocyte survival and B cell lymphomagenesis
Source: FEBS J. 2018 Mar 24;285(8):1403–18. doi: 10.1111/febs.14426 (PMC5947286; doi:10.1111/febs.14426)
Supplement: Supplementary file 1 — Table S1. Phenotypic characterization of lymphomas arising in Eμ‐MYC TG mice. Table S2. Phenotypic characterization of lymphomas arising in Eμ‐MYC/Vav‐BFL1 DT mice. Table S3. Phenotypic characterization of lymphomas arising in Eμ‐MYC/Vav‐BCLX DT mice. [file FEBS-285-1403-s001.pdf › febs14426-sup-0001-TableS1-3.pdf]

FEBS PRESS
science publishing by scientists

WILEY

The FEBS Journal

www.febsjournal.org

# Differential effects of Vav-promoter-driven overexpression of BCLX and BFL1 on lymphocyte survival and B cell lymphomagenesis

Selma Tuzlak, Manuel D. Haschka, Anna-Maria Mokina, Thomas Rülicke, Suzanne Cory, Verena Labi and Andreas Villunger

DOI: 10.1111/febs.14426

**Table S1. Phenotypic characterization of lymphomas arising in *Eµ-MYC* TG mice.** Diseased *Eµ-Myc* TG mice were bled from the submandibular vein and bone marrow (BM), spleen, thymus, mesenteric lymph nodes (mLN) and peripheral lymph nodes (pLN, consisting of mandibular, axillary and inguinal LN) were isolated after sacrifice. Single cell suspensions were analyzed by flow cytometry for the expression of B220, CD4, CD19, and IgM. Total $CD19^{-}B220^{+}CD4^{+}$, and $CD19^{+}B220^{+}CD4^{-}$ lymphoma cells were quantified. $CD19^{+}B220^{+}CD4^{-}$ lymphoma cells were further characterized into $IgM^{+}$ and $IgM^{-}$ cells and thereby the phenotype of the lymphoma in the respective organ was determined. Mixed phenotype refers to the presence of both $CD19^{+}B220^{+}IgM^{+}$ and $CD19^{+}B220^{+}IgM^{-}$ populations. The overall phenotype refers to the cumulative results of all organs from a respective mouse and is depicted in Fig. 5E. Furthermore, spleen weights, thymus weights, white blood cell (WBC) counts and tumor onset are indicated.

# Tuzlak et al. Table S1

| genotype | mouse # | organ | % total CD19-B220+CD4+ | % B cells (CD19+B220+) | % IgM+ of B cells | % IgM- of B cells | phenotype | overall phenotype | spleen weight [g] | thymus weight [g] | WBC count [10^3/mm^3] | tumor onset [d] |
|---|---|---|---|---|---|---|---|---|---|---|---|---|
| **MYC** | A2 | BM | 0,1 | 55,3 | 0,25 | 99,5 | IgM- | **IgM-** | 0,42 | 0,15 | 44,7 | 91 |
| | | blood | 0,031 | 51,8 | 5,11 | 94,8 | IgM- | | | | | |
| | | spleen | 0,042 | 49,8 | 5,27 | 94,4 | IgM- | | | | | |
| | | thymus | 0 | 32,1 | 0,49 | 99,5 | IgM- | | | | | |
| | | mLN | 0,045 | 84,5 | 2,02 | 98 | IgM- | | | | | |
| | | pLN | 0,019 | 77,9 | 1,29 | 98,7 | IgM- | | | | | |
| | A15 | BM | 0,016 | 77,1 | 0,041 | 100 | IgM- | **IgM-** | 0,37 | 0,08 | 10,1 | 101 |
| | | blood | 9,79E-03 | 41,5 | 0,19 | 99,8 | IgM- | | | | | |
| | | spleen | 8,16E-03 | 31,7 | 0,19 | 99,8 | IgM- | | | | | |
| | | thymus | 0,11 | 43,2 | 0,17 | 99,8 | IgM- | | | | | |
| | | mLN | 0,018 | 71,6 | 0,05 | 99,9 | IgM- | | | | | |
| | | pLN | 9,69E-03 | 77,7 | 0,081 | 99,9 | IgM- | | | | | |
| | A17 | BM | 0,45 | 33,9 | 0,42 | 99,6 | IgM- | **IgM-** | 0,29 | 0,07 | 9,8 | 129 |
| | | blood | 0,01 | 58,4 | 4,09 | 95,8 | IgM- | | | | | |
| | | spleen | 0,041 | 47,2 | 5,7 | 94,1 | IgM- | | | | | |
| | | thymus | 0,041 | 2,39 | 5,89 | 93,4 | IgM- | | | | | |
| | | mLN | 0,064 | 70,1 | 7,84 | 92 | IgM- | | | | | |
| | | pLN | 0,11 | 70,3 | 8,26 | 91,6 | IgM- | | | | | |
| | A34 | BM | 1,24 | 55,7 | 13,4 | 85,4 | IgM- | **IgM-** | 0,09 | 0,12 | 6,2 | 42 |
| | | blood | 5,04E-03 | 21,8 | 24,8 | 73,7 | IgM- | | | | | |
| | | spleen | 0,17 | 29,7 | 32,5 | 65,8 | IgM- | | | | | |
| | | thymus | 0,36 | 78,9 | 3,17 | 96,2 | IgM- | | | | | |
| | | mLN | 0,26 | 92,4 | 3,5 | 95,9 | IgM- | | | | | |
| | | pLN | 0 | 15 | 20,5 | 79,5 | IgM- | | | | | |
| | A47 | BM | 0,33 | 39,5 | 22,8 | 74,5 | IgM- | **mixed** | 0,56 | 0,24 | 44,5 | 53 |
| | | blood | 0,01 | 37,6 | 17,6 | 80,6 | IgM- | | | | | |
| | | spleen | 0,013 | 56,6 | 22,1 | 77,9 | IgM- | | | | | |
| | | thymus | 0,028 | 40,9 | 44 | 56 | mixed | | | | | |
| | | mLN | 0,035 | 77,3 | 36,9 | 61,5 | mixed | | | | | |
| | | pLN | 0,1 | 80,7 | 39,8 | 56,7 | mixed | | | | | |
| | A21 | BM | 0,23 | 42,5 | 0,96 | 98,9 | IgM- | **IgM-** | 0,2 | 0,09 | 13,8 | 171 |
| | | blood | 3,13E-03 | 28,7 | 9,92 | 89,6 | IgM- | | | | | |
| | | mLN | 0,058 | 82,6 | 2,21 | 97,6 | IgM- | | | | | |
| | | pLN | 0,13 | 83,3 | 3,56 | 96,2 | IgM- | | | | | |
| | | spleen | 0,055 | 47,8 | 7,02 | 92,1 | IgM- | | | | | |
| | | thymus | 0,034 | 4,08 | 2,29 | 97,4 | IgM- | | | | | |
| | A28 | BM | 0,048 | 75,4 | 4,32 | 94,8 | IgM- | **IgM-** | 0,73 | 0,07 | 13,2 | 139 |
| | | blood | 0,013 | 37,2 | 21,9 | 77,1 | IgM- | | | | | |
| | | mLN | 0,034 | 83,7 | 11,8 | 87,6 | IgM- | | | | | |
| | | pLN | 0,041 | 81,2 | 10,5 | 88,9 | IgM- | | | | | |
| | | spleen | 0,084 | 47,4 | 29,9 | 69,3 | IgM- | | | | | |
| | | thymus | 0,069 | 24,2 | 17 | 82,1 | IgM- | | | | | |
| | A37 | BM | 0 | 92,9 | 0,23 | 99,5 | IgM- | **IgM-** | 0,44 | 0,15 | 13,2 | 134 |
| | | blood | 0 | 39,7 | 6,49 | 93,2 | IgM- | | | | | |
| | | mLN | 0,1 | 65,8 | 7,83 | 91,7 | IgM- | | | | | |
| | | pLN | 0,11 | 57,8 | 4,1 | 95,5 | IgM- | | | | | |
| | | spleen | 0,11 | 36,2 | 6,88 | 92,7 | IgM- | | | | | |
| | | thymus | 0,11 | 75,7 | 1,1 | 98,5 | IgM- | | | | | |
| | B76 | BM | 0,4 | 48,3 | 0,45 | 99,5 | IgM- | **IgM-** | 0,74 | 0,26 | 77,8 | 73 |
| | | blood | 0,025 | 67,1 | 3,17 | 96,8 | IgM- | | | | | |
| | | spleen | 6,00E-03 | 31,9 | 3,61 | 96,4 | IgM- | | | | | |
| | | thymus | 0,032 | 42,6 | 0,34 | 99,7 | IgM- | | | | | |
| | | mLN | 0,1 | 75,3 | 3,1 | 97,1 | IgM- | | | | | |
| | | pLN | 0,041 | 66 | 2,88 | 97,3 | IgM- | | | | | |
| | B74 | BM | 0,5 | 47,7 | 0,77 | 99,2 | IgM- | **IgM-** | 0,48 | 0,24 | 96,5 | 80 |
| | | blood | 0,058 | 77,3 | 3,07 | 96,8 | IgM- | | | | | |
| | | spleen | 0,027 | 44,4 | 4,29 | 95,9 | IgM- | | | | | |
| | | thymus | 0,06 | 74 | 0,17 | 99,8 | IgM- | | | | | |
| | | mLN | 0,044 | 80,3 | 3,6 | 96,3 | IgM- | | | | | |
| | | pLN | 0,12 | 67,2 | 5,77 | 94,1 | IgM- | | | | | |

**Table S2. Phenotypic characterization of lymphomas arising in *Eμ-MYC/Vav-BFL1* DT mice.** Diseased *Eμ-Myc/Vav-BFL1* DT mice were bled from the submandibular vein and bone marrow (BM), spleen, thymus, mesenteric lymph nodes (mLN) and peripheral lymph nodes (pLN, consisting of mandibular, axillary and inguinal LN) were isolated after sacrifice. Single cell suspensions were analyzed by flow cytometry for the expression of B220, CD4, CD19, and IgM. Total $CD19^{-}B220^{+}CD4^{+}$, and $CD19^{+}B220^{+}CD4^{-}$ lymphoma cells were quantified. $CD19^{+}B220^{+}CD4^{-}$ lymphoma cells were further characterized into $IgM^{+}$ and $IgM^{-}$ cells and thereby the phenotype of the lymphoma in the respective organ was determined. Mixed phenotype refers to the presence of both $CD19^{+}B220^{+}IgM^{+}$ and $CD19^{+}B220^{+}IgM^{-}$ populations. The overall phenotype refers to the cumulative results of all organs from a respective mouse and is depicted in Fig. 5E. Furthermore, spleen weights, thymus weights, white blood cell (WBC) counts and tumor onset are indicated.

# Tuzlak et al. Table S2

| genotype | mouse # | organ | % total CD19-B220+CD4+ | % B cells (CD19+B220+) | % IgM+ of B cells | % IgM- of B cells | phenotype | overall phenotype | spleen weight [g] | thymus weight [g] | WBC count [10^3/mm^3] | tumor onset [d] |
|---|---|---|---|---|---|---|---|---|---|---|---|---|
| **MYC/BFL1** | A23 | BM | 4,77 | 47,1 | 4,51 | 95,4 | IgM- | **IgM- early CD4** | 0,23 | 0,37 | 72,6 | 49 |
| | | blood | 1,89 | 76 | 9,25 | 90,6 | IgM- | | | | | |
| | | spleen | 0 | 53,8 | 13,3 | 86,5 | IgM- | | | | | |
| | | thymus | **37,2** | 40,6 | 2,19 | 97,7 | IgM- | | | | | |
| | | mLN | 0,35 | 94,2 | 0,32 | 99,6 | IgM- | | | | | |
| | | pLN | 0 | 61 | 9,24 | 90,5 | IgM- | | | | | |
| | A40 | BM | 0,032 | 85,8 | 1,76 | 98,2 | IgM- | **IgM-** | 0,37 | 0,34 | 138 | 51 |
| | | blood | 8,89E-03 | 55,6 | 5,05 | 94,9 | IgM- | | | | | |
| | | spleen | 6,35E-03 | 63 | 7,97 | 91,9 | IgM- | | | | | |
| | | thymus | 0,024 | 91,8 | 18,3 | 81,4 | IgM- | | | | | |
| | | mLN | 0,021 | 63 | 1,64 | 98,3 | IgM- | | | | | |
| | | pLN | 0,15 | 74,1 | 12,9 | 87 | IgM- | | | | | |
| | A52 | BM | 3,32 | 58,1 | 29,8 | 69,9 | mixed | **mixed early CD4** | 0,38 | 0,34 | 92 | 53 |
| | | blood | 0,39 | 81,4 | 58,3 | 41,5 | mixed | | | | | |
| | | spleen | 0,31 | 67,2 | 74,6 | 24,3 | mixed | | | | | |
| | | thymus | **34,2** | 44,4 | 29,4 | 69,9 | mixed | | | | | |
| | | mLN | **11,8** | 58,1 | 46,6 | 52,1 | mixed | | | | | |
| | | pLN | 9,4 | 70 | 39,7 | 58,6 | mixed | | | | | |
| | A45 | spleen | 0,015 | 36,6 | 33,7 | 65,3 | mixed | **mixed** | 0,43 | 0,85 | 382,5 | 53 |
| | | thymus | 0,024 | 93,6 | 31,8 | 65,8 | mixed | | | | | |
| | | pLN | 0,063 | 84 | 12,6 | 86,2 | IgM- | | | | | |
| | | BM | 0,28 | 63,4 | 31,1 | 67,8 | mixed | | | | | |
| | | blood | 0,025 | 40,5 | 31,8 | 68,1 | mixed | | | | | |
| | A24 | BM | 0,54 | 56,8 | 0,82 | 99,1 | IgM- | **IgM- early CD4** | 0,53 | 0,29 | 363 | 55 |
| | | blood | 0,036 | 27,6 | 0,77 | 99,2 | IgM- | | | | | |
| | | spleen | 0,021 | 37 | 1,45 | 98,5 | IgM- | | | | | |
| | | thymus | **11** | 53,7 | 0,31 | 99,7 | IgM- | | | | | |
| | | mLN | 0,78 | 82,4 | 1,12 | 98,8 | IgM- | | | | | |
| | | pLN | 0,51 | 81,4 | 0,6 | 99,4 | IgM- | | | | | |
| | A38 | BM | 1,07 | 63,4 | 4,23 | 95,5 | IgM- | **IgM- early CD4** | 0,12 | 0,18 | 4,5 | 37 |
| | | blood | 0,098 | 37,1 | 20,5 | 79,1 | IgM- | | | | | |
| | | spleen | 0,19 | 28,1 | 22,3 | 69,6 | IgM- | | | | | |
| | | thymus | **25,3** | 45,8 | 6,04 | 92,7 | IgM- | | | | | |
| | | mLN | 0,47 | 80,8 | 1,43 | 98,2 | IgM- | | | | | |
| | | pLN | 1,59 | 87,2 | 6,02 | 92,1 | IgM- | | | | | |
| | A48 | BM | 1,32 | 59,9 | 2,59 | 97,1 | IgM- | **IgM- early CD4** | 0,49 | 0,29 | 194 | 39 |
| | | blood | 0,066 | 34,2 | 8,06 | 91,5 | IgM- | | | | | |
| | | thymus | **10,2** | 67,8 | 7,17 | 91,6 | IgM- | | | | | |
| | | mLN | 0,97 | 85,3 | 9,27 | 89 | IgM- | | | | | |
| | | pLN | 2,33 | 84,1 | 11,9 | 86,8 | IgM- | | | | | |
| | A56 | BM | 0,3 | 68,2 | 0,45 | 99,5 | IgM- | **IgM- early CD4** | 0,44 | 0,31 | 127,5 | 47 |
| | | blood | 0,038 | 52,2 | 1,49 | 98,5 | IgM- | | | | | |
| | | spleen | 0,036 | 51,4 | 3,69 | 96,2 | IgM- | | | | | |
| | | thymus | **8,01** | 82,1 | 0,49 | 99,5 | IgM- | | | | | |
| | | mLN | 1,29 | 81,4 | 1,77 | 98,2 | IgM- | | | | | |
| | | pLN | 2,39 | 76,4 | 3,02 | 96,9 | IgM- | | | | | |
| | A8 | BM | 2,94E-03 | 67,9 | 88,7 | 10,8 | IgM+ | **IgM+** | 0,24 | 0,45 | 79,5 | 61 |
| | | spleen | 0 | 54,9 | 96,2 | 1,96 | IgM+ | | | | | |
| | | thymus | 0 | 97,1 | 96,9 | 2,48 | IgM+ | | | | | |
| | | mLN | 0 | 87 | 51,9 | 45 | IgM+ | | | | | |
| | | pLN | 0 | 96,1 | 97,2 | 1,73 | IgM+ | | | | | |
| | A9 | BM | 1,81 | 69,8 | 0,54 | 99,4 | IgM- | **IgM- early CD4** | 0,67 | 0,32 | 38 | 61 |
| | | spleen | 0,39 | 82,6 | 2,35 | 97,3 | IgM- | | | | | |
| | | thymus | **52,1** | 30 | 3,22 | 96,3 | IgM- | | | | | |
| | | mLN | **21,6** | 48,2 | 6,18 | 92,9 | IgM- | | | | | |
| | | pLN | 1,13 | 94,1 | 0,37 | 99,5 | IgM- | | | | | |
| | A13 | BM | 0,098 | 79 | 11,2 | 88,3 | IgM- | **IgM- early CD4** | 0,17 | 0,17 | 17,6 | 62 |
| | | spleen | 0,057 | 70,3 | 28,3 | 71,4 | IgM- | | | | | |
| | | thymus | **6,04** | 88,3 | 3,33 | 96,5 | IgM- | | | | | |
| | | mLN | 0,91 | 89,8 | 4,87 | 95 | IgM- | | | | | |
| | | pLN | 2,99 | 88,8 | 6,7 | 93 | IgM- | | | | | |

**Table S3. Phenotypic characterization of lymphomas arising in *Eμ-MYC/Vav-BCLX* DT mice** Diseased *Eμ-Myc/Vav-BCLX* DT mice were bled from the submandibular vein and bone marrow (BM), spleen, thymus, mesenteric lymph nodes (mLN) and peripheral lymph nodes (pLN, consisting of mandibular, axillary and inguinal LN) were isolated after sacrifice. Single cell suspensions were analyzed by flow cytometry for the expression of B220, CD4, CD19, and IgM. Total $CD19^{-}B220^{+}CD4^{+}$, and $CD19^{+}B220^{+}CD4^{-}$ lymphoma cells were quantified. $CD19^{+}B220^{+}CD4^{-}$ lymphoma cells were further characterized into $IgM^{+}$ and $IgM^{-}$ cells and thereby the phenotype of the lymphoma in the respective organ was determined. Mixed phenotype refers to the presence of both $CD19^{+}B220^{+}IgM^{+}$ and $CD19^{+}B220^{+}IgM^{-}$ populations. The overall phenotype refers to the cumulative results of all organs from a respective mouse and is depicted in Fig. 5E. Furthermore, spleen weights, thymus weights, white blood cell (WBC) counts and tumor onset are indicated.

# Tuzlak et al. Table S3

| genotype | mouse # | organ | % total CD19-B220+CD4+ | % B cells (CD19+B220+) | % IgM+ of B cells | % IgM- of B cells | phenotype | overall phenotype | spleen weight [g] | thymus weight [g] | WBC count [10^3/mm^3] | tumor onset [d] |
|---|---|---|---|---|---|---|---|---|---|---|---|---|
| **MYC/BCLX** | A70 | BM | 1,01 | 45 | 45,9 | 53,2 | mixed | **mixed** | 0,49 | 0,51 | 122 | 58 |
| | | blood | 0,019 | 64 | 73 | 26 | IgM+ | | | | | |
| | | spleen | 1,70E-02 | 49,5 | 81,6 | 15,8 | IgM+ | | | | | |
| | | thymus | 0,07 | 55,4 | 3,13 | 95,8 | IgM- | | | | | |
| | | mLN | 0,32 | 55,2 | 50,1 | 46 | mixed | | | | | |
| | | pLN | 0,23 | 56,1 | 36,5 | 60,2 | mixed | | | | | |
| | A74 | BM | 0,49 | 52,3 | 1,37 | 98,4 | IgM- | **IgM-** | 0,62 | 0,33 | 74,5 | 58 |
| | | blood | 0,015 | 73,7 | 4,45 | 95,2 | IgM- | | | | | |
| | | spleen | 1,50E-02 | 47,3 | 4,45 | 94,8 | IgM- | | | | | |
| | | thymus | 0,056 | 50,6 | 0,9 | 98,9 | IgM- | | | | | |
| | | mLN | 0,26 | 63,1 | 6,74 | 91,8 | IgM- | | | | | |
| | | pLN | 0,035 | 62,3 | 2,68 | 96,7 | IgM- | | | | | |
| | A75 | BM | 0,25 | 53,4 | 95,1 | 4,64 | IgM+ | **IgM+** | 0,39 | 0,55 | 145 | 58 |
| | | blood | 0,01 | 80,7 | 97 | 2,73 | IgM+ | | | | | |
| | | spleen | 2,65E-03 | 43 | 96 | 2,94 | IgM+ | | | | | |
| | | thymus | 2,10E-02 | 55,9 | 84,5 | 12 | IgM+ | | | | | |
| | | mLN | 0,17 | 44,1 | 77,9 | 20,3 | IgM+ | | | | | |
| | | pLN | 0,2 | 51,7 | 81,4 | 16,7 | IgM+ | | | | | |
| | A55 | BM | 0,53 | 45,5 | 18,8 | 80,8 | IgM- | **mixed early CD4** | 0,21 | 0,04 | 33,1 | 119 |
| | | blood | 0,083 | 70,4 | 83,9 | 13,8 | IgM+ | | | | | |
| | | spleen | 0,18 | 52,6 | 71 | 23,2 | IgM+ | | | | | |
| | | thymus | **15,7** | 2,21 | 61,3 | 37,3 | mixed | | | | | |
| | | mLN | 0,81 | 65,6 | 27 | 66,1 | IgM- | | | | | |
| | | pLN | 0,21 | 54,4 | 8,19 | 90,6 | IgM- | | | | | |
| | A7 | BM | 3,13 | 30,8 | 76,8 | 22,4 | IgM+ | **IgM+ early CD4** | 0,7 | 0,84 | 497 | 72 |
| | | blood | 2,54 | 78,9 | 27,4 | 71,7 | IgM+ | | | | | |
| | | spleen | 0,66 | 58,4 | 97,3 | 2,74 | IgM+ | | | | | |
| | | thymus | **8,84** | 87,8 | 91,9 | 8,23 | IgM+ | | | | | |
| | | mLN | **20,1** | 36,8 | 79,1 | 17 | IgM+ | | | | | |
| | | pLN | **12,4** | 46,5 | 82,3 | 16,6 | IgM+ | | | | | |
| | A8 | BM | 0,61 | 52,4 | 45,4 | 54,3 | mixed | **mixed** | 0,48 | 0,21 | 78 | 78 |
| | | blood | 0,026 | 62,5 | 74,5 | 26,1 | IgM+ | | | | | |
| | | spleen | 0,024 | 46,2 | 77,1 | 22,2 | IgM+ | | | | | |
| | | thymus | 0,48 | 76,4 | 92,7 | 6,66 | IgM+ | | | | | |
| | | mLN | 0,15 | 60,7 | 27,4 | 71,9 | IgM- | | | | | |
| | | pLN | 0,12 | 69,1 | 24,7 | 74,6 | IgM- | | | | | |
| | A52 | BM | 0,25 | 44,3 | 94,4 | 5,34 | IgM+ | **IgM+** | 0,38 | 0,99 | 210,5 | 62 |
| | | blood | 9,45E-03 | 86,1 | 33,3 | 64,5 | IgM+ | | | | | |
| | | spleen | 9,10E-03 | 34,6 | 91,6 | 8,26 | IgM+ | | | | | |
| | | thymus | 4,88E-03 | 93,2 | 95,5 | 4,25 | IgM+ | | | | | |
| | | mLN | 0,16 | 39,6 | 77,3 | 21,6 | IgM+ | | | | | |
| | | pLN | 0,16 | 57,2 | 91,9 | 7,68 | IgM+ | | | | | |
| | A50 | BM | 0,87 | 57,9 | 0,73 | 99,2 | IgM- | **IgM-** | 0,36 | 0,11 | 29 | 62 |
| | | blood | 0,05 | 72,3 | 14,3 | 84,4 | IgM- | | | | | |
| | | spleen | 0,068 | 62,1 | 10,8 | 88,6 | IgM- | | | | | |
| | | thymus | 0,15 | 2,12 | 4,82 | 94,9 | no tumor | | | | | |
| | | mLN | 0,14 | 65,4 | 12,2 | 87,8 | IgM- | | | | | |
| | | pLN | 0,15 | 61,5 | 10,7 | 89,7 | IgM- | | | | | |
| | A59 | BM | 0,28 | 66,9 | 97,5 | 2,3 | IgM+ | **IgM+** | 0,64 | 0,62 | 361,5 | 124 |
| | | blood | 0,042 | 92,1 | 99,2 | 0,73 | IgM+ | | | | | |
| | | spleen | 0,028 | 70,3 | 97,8 | 1,65 | IgM+ | | | | | |
| | | thymus | 2,44 | 90,2 | 95,6 | 3,58 | IgM+ | | | | | |
| | | mLN | 2,14 | 79,9 | 56,4 | 41 | mixed | | | | | |
| | | pLN | 0,71 | 88,2 | 63,1 | 31,5 | mixed | | | | | |
